# Reference Gene Selection for Quantitative Real-Time RT-PCR Normalization in the Half-Smooth Tongue Sole (*Cynoglossus semilaevis*) at Different Developmental Stages, in Various Tissue Types and on Exposure to Chemicals

**DOI:** 10.1371/journal.pone.0091715

**Published:** 2014-03-25

**Authors:** Conghui Liu, Nian Xin, Yi Zhai, Liming Jiang, Jieming Zhai, Quanqi Zhang, Jie Qi

**Affiliations:** 1 Key Laboratory of Marine Genetics and Breeding, Ocean University of China, Qingdao, Shandong, China; 2 Laizhou Mingbo Aquatic CO., Ltd., Laizhou, Shandong, China; Ludwig-Maximilians-University Munich, Germany

## Abstract

Quantitative real time RT-PCR has been described as the most sensitive method for the detection of low abundance mRNA. To date, no reference genes have been screened in the half-smooth tongue sole (*Cynoglossus semilaevis*). The aim of this study was to select the most stable genes for quantitative real-time RT-PCR. Eight housekeeping genes (18S, TUBA, B2M, ACTB, EF1A, GAPDH, RPL17 and UBCE) were tested at different developmental stages, in different tissues, and following exposure to the drug SB-431542. Using geNorm, BestKeeper and NormFinder software, GAPDH/B2M, GAPDH/18S and UBCE/GAPDH were identified as the most suitable genes from samples taken of different developmental stages while 18S/RPL17 were consistently ranked as the best reference genes for different tissue types. Furthermore, TUBA/B2M, TUBA/UBCE and B2M/TUBA were found to be the most suitable genes in samples treated with the drug, SB-431542 by geNorm, BestKeeper and NormFinder respectively. Across both different developmental stages and tissue types, the combination of 18S and GAPDH was the most stable reference gene analyzed by Ref-Finder. To test and verify the screened reference genes, the expression profiles of LEFTY-normalized to the combination of GAPDH/18S and ACTB were presented. These results will be useful for future gene-expression studies in the half-smooth tongue sole.

## Introduction

Quantitative real-time PCR (qRT-PCR) plays an extremely important role in studies on gene expression and determination of complex molecular pathways in various biological systems [1]. It is a robust method with obvious advantages, including sensitivity, large dynamic range, and the potential for high throughput and accurate quantification [2]. The read-out can be either an absolute number of copies, or a relative amount normalized to an internal control gene [2]. The accuracy of this technique, however, might be affected at multiple stages throughout the experimental process and by several factors. It has been proposed that housekeeping genes might be used as reference genes for normalizing the results of qRT-PCR because of their consistent expression, both with respect to different developmental stages and exposure to different environmental factors. In reality, however, no single reference gene can be used for longitudinal studies for different tissues, ontogenetic stages and experimental conditions. The transcript levels of genes deemed as housekeeping traditionally may also vary considerably [3]. Thus, the optimal genes can only be confirmed with an analysis of models based on particular cases. An accepted method used to reduce errors to a minimum is to normalize the target gene to a set of reference genes identified through an analysis of parameter models [1], [4]. GeNorm, BestKeeper and NormFinder are Visual Basic Applications that were developed for running in Microsoft Excel and they are widely used in various conditional experiments [4]. Pairwise variation is calculated by geNorm to determine the most stable reference genes, whereas BestKeeper is used to evaluate the coefficient of variation. NormFinder takes the analysis of intra- and inter-group variations for normalization into consideration.

The half-smooth tongue sole (*Cynoglossus semilaevis*) is an important farmed marine fish, which shows behavioral and morphological left-right asymmetry, with one eye migrating to the opposite side (metamorphosis) during the larval stages [5]. A high larval mortality due to starvation can occur during metamorphosis when body malformations cause the mouth to fail to close. Whereas the mechanism regulating this external asymmetry remains largely unknown, evidence suggests that the Nodal signaling pathway is involved in the eye laterality of a flatfish similar to the sole, the Japanese flounder [6]–[9]. More studies are needed to analyze the expression profiles of genes related to the Nodal signaling pathway. Currently, little work has been conducted on the selection of reference genes in the half-smooth tongue sole, although some reference genes have been identified in the Atlantic halibut and Japanese flounder [10]–[12]. While it is useful to consider the reference genes identified from the halibut and flounder, inter-species variation can occur and so it is more accurate to identify the particular genes for the species under investigation, in this case the half-smooth tongue sole [13].

The aim of this work was to screen optimal sets of reference genes for 18 different developmental stages, 8 tissue types and following treatment with SB-431542, an inhibitor of the Nodal signaling pathway. Three statistical algorithms, geNorm, BestKeeper and NormFinder, were used to analyze the qRT-PCR data. In addition, the ontogenesis expression profiles of LEFTY were investigated to confirm the efficacy of the selected reference genes as this is reported to be part of the Nodal-pathway that plays a role in controlling eye laterality in the flounder [7], as well as being shown to be expressed on the left side of the dorsal diencephalon and internal organs to form fixed laterality [6], [8]. The results obtained in this study provide an essential tool for future gene expression studies in the half-smooth tongue sole.

## Materials and Methods

### Ethics Statement

The half-smooth tongue sole used in this study were collected from local aquatic farms under a permit from the local government of Yantai, Shandong, China. All handling of the half-smooth tongue sole was conducted in accordance with the guidelines and regulations established by the Ocean University of China and the local government of YanTai.

### Fish Rearing and Sample Collection

Fertilized eggs were obtained by artificial fertilization and then maintained at 22°C. The developmental stages were determined by observation via microscopy. A total of 18 developmental stages were selected:1-cell (0.5 hours post fertilization; hpf), 4-cell (1.5 hpf), 8-cell (1.8 hpf), 16-cell (2.3 hpf), 32-cell (2.8 hpf), 1k-cell (6 hpf) and sphere (11 hpf) stages, as well as 30% epiboly (13.5 hpf), and the 2-somite (20 hpf), 15-somite (3.5 hpf), 21-somite (27.5 hpf), 27-somite (30 hpf) and metamorphosis stages D, E, F, G, H, and I, as described by Minami (1982) [14]. In brief, the metamorphosis stages can be described as follows: Stage D (the stage prior to the start of eye migration, 15 DAH; day after hatching); Stage E (the eye begins to migrate, 17 DAH); Stage F (the migrating eye is visible from the ocular side, 18 DAH); Stage G (the upper edge of the migrating eye is beyond the dorsal margin, 19 DAH); Stage H (the upper edge of the migrating eye is beyond the dorsal mid-line, 20 DAH); Stage I (the entire migrating eye is past the dorsal mid-line, 22 DAH) (Minami, 1982). The larvae were raised at 22°C.

These 18 stages were arranged into 3 groups each containing 6 stages, called developmental stage I (from the 1-cell to 1k-cell stage), II (sphere stage to 27-somite stage) and III (metamorphosis stages D-I). For developmental stages I and II, 30 embryos were used per stage, while for developmental stage III, three larvae were used. In chemical treated experiments, embryos were treated from the 16-cell stage to the 21-somite stage with SB-431542 at 100 μM and 200 μM (or with double-distilled water or DMSO as controls) with 30 embryos being treated per experimental group. All experiments were performed in biological triplicate.

Regarding the different tissues, a total of six adult fish (i.e., three female and three male fish) were involved in this investigation. Eggs and sperm came from three female and three male fish, respectively, while the other tissues (muscle, kidney, gill, spleen, liver, intestines, egg and sperm) were selected from 3 different adult fish randomly, and again experiments were performed in triplicate, and then stored in liquid nitrogen immediately until use. All procedures complied with the Institutional Animal Care and Use Committee of Ocean University of China and were specifically approved for this study.

### Selection of Reference Genes

A total of eight candidate genes were selected for the gene expression stability analysis as follows: 18S (Ribosomal RNA), TUBA (α-Tubulin), ACTB (β-actin), B2M (β-2-Microglobulin), EF1A (Elongation factor-1-α), GAPDH (Glyceraldehyde-3-phosphate), RPL17 (Ribosomal protein L17) and UBCE (Ubiquitin-conjugating enzyme). Moreover, LEFTY (Fw: 5′-CCCAAGGTGTTGTCCGTCTG-3′; Rev: 5′-ATTCAAGGCTGATCCCC ATTC-3′) was used to verify the proper reference genes. All the genes were identified from an unpublished cDNA library of *Cynoglossus semilaevis*. The primers were designed with Primer Premier 5.0 software (PREMIER Biosoft International, Palo Alto, CA). The full gene name, function, primers and PCR parameters are shown in Table I. The length of PCR products ranged from 70 to 170 bps, and the mean melting temperature of the forward and reverse primers ranged between 61.0°C and 62.7°C.

### RNA Extraction, cDNA Synthesis and RT-PCR

Total RNA was extracted from samples using Trizol reagent according to the manufacturer’s instructions. The concentration and purity of the total RNA extracted were determined using a Nano Photometer. The absorbance ratio of each sample at OD 260/280 was between 1.9 and 2.0, and the OD 260/230 was approximately 2.0. The integrity of total RNA was assessed via 1% ethidium bromide agarose gel electrophoresis. A total of 1 μg RNA from each sample was reverse transcribed according to the instructions of the PrimeScript™ RT reagent kit with gDNA Eraser (Takara, Japan), and the final volume was 20 μl. Standard RT-PCR was carried out using Premix Ex Taq to characterize all the primer pairs according to the length and specificity of the products.

### Quantitative Real-time PCR (qRT-PCR)

Each 20 μl reaction volume contained 10 μl 2X SYBR Premix Ex Taq™, 0.4 μl ROX Reference Dye II, 0.4 μl of each primer (10 μM), 2 μl 10X diluted cDNA template (1000X diluted cDNA for 18S rRNA) and 6.8 μl nuclease-free water. The PCR conditions were as follows: 95°C for 30s followed by 40 cycles of 95°C for 5s and then 60°C for 40s. PCR was performed using an Applied Biosystems 7500 Real-Time PCR system. Three technical replicates were performed for each sample.

### Data Analysis

To comprehensively and systematically assess the expression variation of the candidate reference genes, all the samples were divided into three broad categories: developmental stages, different tissues, and chemical treatment. The developmental stages were subdivided into three groups: Stage I, the 1-cell stage (i.e., a newly fertilized egg) to the sphere stage; Stage II, 30% epiboly to the 27-somite stage; and Stage III, throughout metamorphosis.

The time sequence fluorescence values (Rn) were collected using the qRT-PCR machine. All raw data, which based on all cDNAs samples of tissue, developmental stage, and chemical-treated, were imported into the PCR Miner software to calculate efficiency (E), cycle threshold (Ct), and Pearson’s coefficients of determination (R^2^) of each reference gene. The expression stability level was determined with geNorm and NormFinder, which use relative expression values as input data by transformed the Ct values to linear scale expression quantities using the 2^-delta-Ct^ method, and BestKeeper based on. the raw Ct value directly using the comparative ΔCT method.

The overall final ranking of reference genes across all developmental stages and tissue types was calculated using Ref-Finder, which integrates the current major computational programs (geNorm, Normfinder, BestKeeper, and the comparative delt Ct method) to compare and rank the stability of candidate reference genes with the geometric mean of individual gene appropriate weight [15].

## Results

### Expression Profiles of Candidate Reference Genes

**Figure 1:**
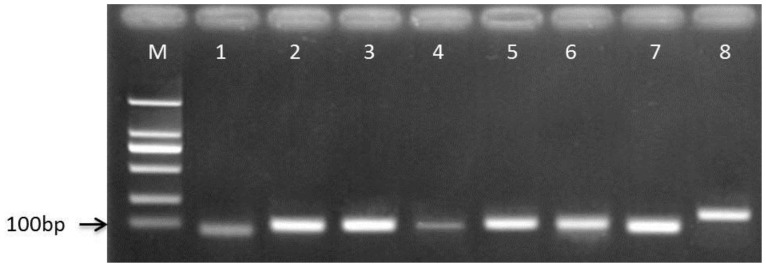
Specificity of qRT-PCR and amplicon length. Amplified fragments shown on 1% agarose gel electrophoresis with ethidium bromide staining. An identical length of specific PCR products was demonstrated. M: Marker DL2000; Lanes 1–8∶18S, TUBA, B2M, ACTB, EF1A, GAPDH, RPL17 and UBCE, respectively.

Eight candidate reference genes, including 18S, TUBA, B2M, ACTB, EF1A, GAPDH, RPL17 and UBCE, were selected for the evaluation of expression stability with n = 3 experiments containing 30 samples per experiment for developmental stages I and II, and 3 samples per experiment for developmental stage III, as described in the Materials and Methods. The amplification specificity of the primers used in the qRT-PCR was evaluated using agarose gel electrophoresis followed by ethidium bromide (EB) staining. The specificity of the primers producing single amplicons was confirmed by the absence of primer dimers and false priming. Specific PCR products of identical length were identified via electrophoresis (Figure 1) and sequencing results. RT-PCR data analysis showed that the amplification efficiency (E) of the eight reference genes ranged from 0.97 for B2M to 1.03 for GAPDH, the mean coefficient variation (R^2^) ranged from 0.9877 for B2M to 0.9938 for GAPDH, and the mean melting temperature (Tm) of the forward and reverse primers ranged from 61.0°C for GAPDH to 62.7°C for UBCE (Table 1).

**Table 1:** Reference genes for gene expression normalization in *Cynoglossus semilaevis.*

Gene	Molecular function	Primer (5′-3′)	Product	E	R^2^	Average T_m_(°C)
symbol			size (bp)			
18S	Ribosomal subunit	Fw: GGTAACGGGGAATCAGGGT	70	0.98	0.989	61.2
		Rv: TGCCTTCCTTGGATGTGGT				
TUBA	Cytoskeletal protein,	Fw: ATCCTCCTGCTGTTGCCTTTG	107	0.99	0.990	62.0
	microtubule activity	Rv: TGACCTGATGTCGTATCGCCTC				
ACTB	Cytoskeletal structural	Fw: GAAATCGCCGCACTTGTTGT	111	1.00	0.987	61.6
	protein	Rv: GGGTCAGGATACCTCTCTTGCTCT				
B2M	Subunit of the MHC class	Fw: TGTTCGTCGTTCTGCCGTGT	112	0.97	0.990	62.2
	I molecule	Rv: TCAGGGTGTTGGGCTTGTTGT				
EF1A	Protein synthesis	Fw: AGGCTGGTATCTCCAAGAACGG	122	0.98	0.987	62.5
		Rv: TCTGGCTGTAAGGGGGCTCA				
GAPDH	Glycolytic enzyme	Fw: GAAGGGCATTCTGGGATACACT	119	1.03	0.993	61.0
	phosphorylating activity	Rv: TCAAAGATGGAGGAGCGGC				
RPL17	Structural constituent of	Fw: AGTGCGTCCCGTTCCGTC	114	1.00	0.991	61.5
	ribosomal subunit	Rv: TCAGCGTTGCTCTCTGCGTT				
UBCE	Ligase activity, protein	Fw: GCAATGGTAGCATCTGCCTCG	170	1.00	0.989	62.7
	degradation	Rv:GCTTCTTCGTTATACTTCTGTCGGTC				

Note: E means reaction efficiencies and R^2^ means Pearson’s coefficients of determination. Average Tm represents the mean melting temperature of the forward (Fw) and reverse (Rv) primers. Each sample contains 3 biological replicates.

**Figure 2:**
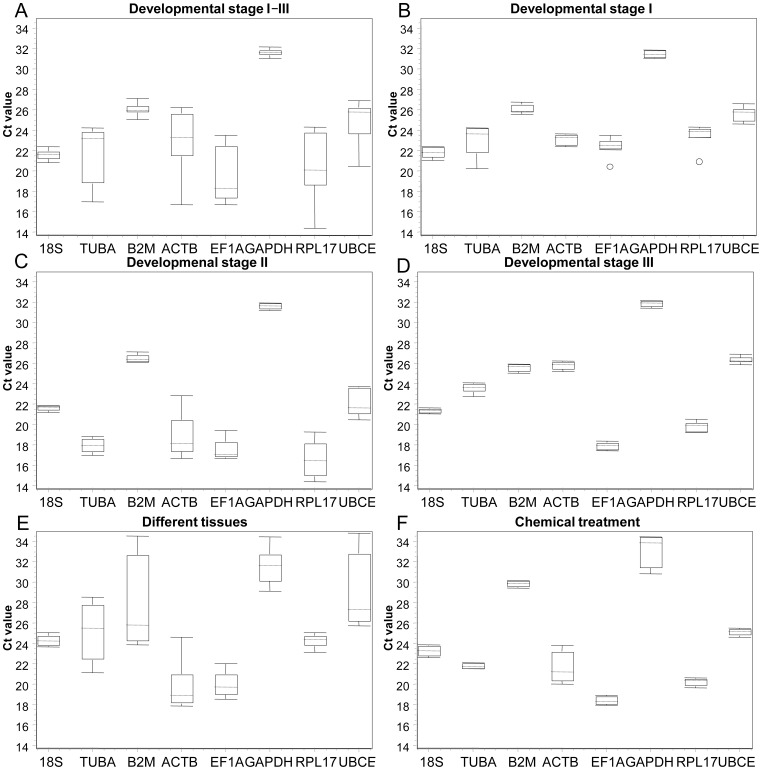
The relative level of mRNA expression of the eight candidate reference genes. The data are presented as mean cycle threshold (Ct) values and shown as box-whisker plots. The boxes represent the inter quartile range of the mean Ct values, while the bars represent the maximum and minimum Ct values. The dotted line represents the mid-hinge and the scattered points represent the abnormal data. (A) Developmental stages I – III; (B) developmental stage I; (C) developmental stage II; (D) metamorphosis stage III; (E) different tissues; (F) chemical treatment.

Throughout all developmental stages (Figure 2A), the genes with the lowest global variability were GAPDH and B2M. GAPDH, ACTB and B2M were the top three genes with the lowest variation in developmental stage I (Figure 2B); 18S, GAPDH and B2M showed little variation in stage II (Figure 2C and Figure A in File S1) and stage III (Figure 2D and Figure B in File S1). However, 18S, RPL17 and EF1A showed smaller variations in different tissues (Figure 2E and Figure C in File S1), and TUBA, B2M and UBCE were the least variable genes in the chemically treated group (Figure 2F and Figure D in File S1).

To assess the Ct value of eight candidate reference genes, we divided the raw expression levels of the genes into three parts according the average Ct values: (1) Genes with a high transcript abundance exhibited an average Ct value below 20; (2) those with median transcript abundance had an average Ct value of 20–25; while (3) those with low transcript abundance had an average Ct value of 25–35. EF1A showed high transcript abundance in all groups except in stage I (all Ct values of eight reference genes were great than 20 in stage I); 18S showed median transcript abundance, while B2M, GAPDH showed low transcript abundance in all sample pools (Table S1). The expression level variations of the eight candidate genes indicated that optimized reference genes could be identified in each group for different research purposes.

### geNorm Analysis

**Figure 3:**
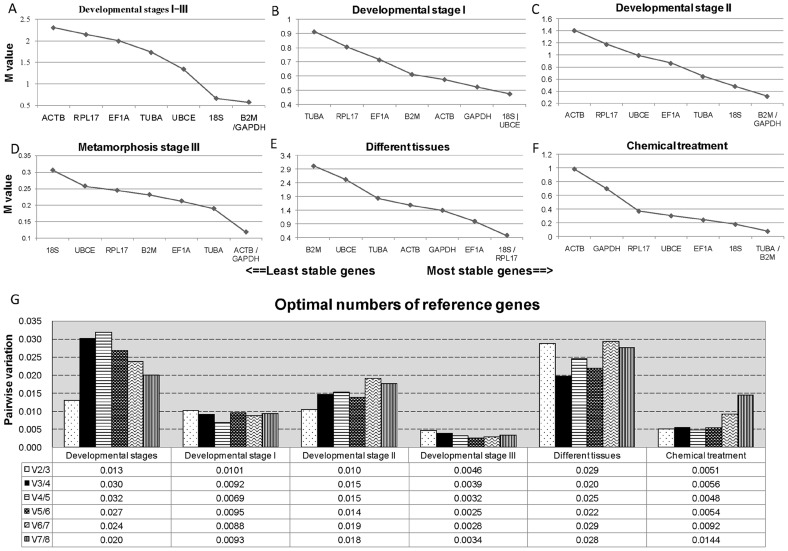
Expression stability (M) analysis and the optimal reference gene number determination with geNorm. The stability values of the eight candidate genes at: (A) Developmental stages I–III; (B) developmental stage I; (C) developmental stage II; (D) metamorphosis stage III; (E) different tissues; (F) chemical treatment; (G) The optimal number of reference genes determined with geNorm, whereby Vn was calculated from leastwise 2 genes. Vn/n+1 present the pairwise variation between 2 sequential Vn and Vn+1.

The geNorm analysis was used to select the best reference genes. Two parameters were considered to quantify reference gene stability: M value (average expression stability) and Vn/n+1 (pairwise variation). Originally, M≤1.5 and Vn/n+1<0.15 are regarded as acceptable levels of expression variability [4]. Recently, an M value below the threshold of 0.5 is generally regarded as being typical for a stable reference gene in a relatively homogeneous sample panel. For more heterogeneous panels, the mean M value may increase to 1 [16]. Depending on the developmental stage, tissue type and chemical treatment, the housekeeping gene expression stability showed some variation. Throughout all developmental stages, the most stable genes were GAPDH and B2M. The M value obtained for these two genes was 0.57, which is below the default limit of M≤1. 18S was also identified as stable, with an M value of 0.66 (Figure 3A). The two most stable genes in developmental stage I were 18S and UBCE (Figure 3B), GAPDH and B2M in stage II (Figure 3C); GAPDH and ACTB in stage III (metamorphic stages) (Figure 3D), 18S and RPL17 for different tissues (Figure 3E) and B2M and TUBA for the chemical treatment group (Figure 3F). To determine the number of required reference genes for each group, the Vn/n+1 was evaluated using geNorm. All Vn/n+1 values, including V2/3, were below 0.15 in each pool (Figure 3G), which indicated that only two genes are needed as an internal control.

### BestKeeper Analysis

**Figure 4:**
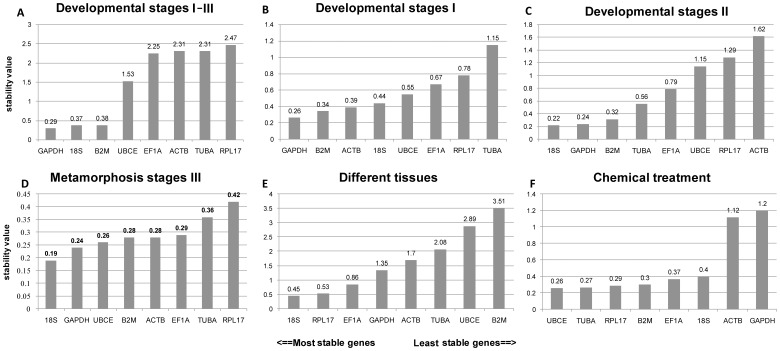
Expression stability analysis of the 8 candidate reference genes by BestKeeper. The stability values of the eight candidate genes at: (A) Developmental stages I–III; (B) developmental stage I; (C) developmental stage II; (D) metamorphosis stage III; (E) different tissues; (F) chemical treatment.

Gene expression variation was calculated for all eight candidate reference genes based on Ct-values and displayed as the standard deviation (SD) and coefficient of variance (CV) by BestKeeper. The lowest values of SD (i.e., usually <1) indicated the highest stability [17]. BestKeeper highlighted GAPDH as the reference gene with the least overall variation from the list of eight candidate genes, with an SD of 0.29, CV value = 0.92 throughout all developmental stages (Figure 4A). The variation in expression of the other candidate reference genes was greater except the 18S and B2M (SD = 0.37, and SD = 0.38 respectively). The order of the candidate reference genes from most stable (lowest SD) to least stable (highest SD) was GAPDH >18S>B2M>UBCE>EF1A>ACTB>TUBA>RPL17. Following for stage I, the ranking was GAPDH>B2M>ACTB >18S>UBCE>EF1A>RPL17> TUBA (Figure 4B); 18S>GAPDH>B2M>TUBA>EF1A>UBCE>RPL17> ACTB for stage II (Figure 4C); and 18S>GAPDH>UBCE>B2M>ACTB>EF1A>TUBA>RPL17 for metamorphic stage III (Figure 4D). For different tissue types, the decreasing order of stability was 18S>RPL17> EF1A>GAPDH>ACTB>TUBA>UBCE>B2M (Figure 4E). 18S (SD = 0.45, CV = 1.86) showed constant expression stability (Table S2); The order of variation changed to UBCE>TUBA>RPL17> B2M>EF1A >18S>ACTB>GAPDH in the chemically treated group (Figure 4F). The most stable gene was UBCE (SD = 0.26, CV = 1.02) (Table S2), and the top five genes had similar stability levels, with SD values ranging from 0.26 to 0.4.

### NormFinder Analysis

NormFinder was applied to validate the most stable expression gene [18]. As shown in Table 2, a high M value represents a high expression variance. Thus, UBCE, GAPDH and 18S occupied the top 3 positions for the most stable expression genes in the group throughout developmental stages I, II and III. The most suitable combination of genes was UBCE and GAPDH (combined stability value = 0.632). The rest were ranked as follows: TUBA, B2M, EF1A, RPL17 and ACTB. Based on developmental stages, the ranking was 18S>GAPDH>UBCE>EF1A>ACTB>RPL17> B2M>TUBA for stage I. The most suitable combination of genes was 18S and GAPDH (combined stability value = 0.154); GAPDH>EF1A >18S>B2M>UBCE>TUBA>RPL17> ACTB for stage II. The most suitable combination of genes was GAPDH and EF1A (combined stability value = 0.203); and GAPDH >18S>B2M>ACTB>UBCE>TUBA>RPL17> EF1A for stage III. The most suitable combination of genes was GAPDH and 18S (combined stability value = 0.038). For different tissue types, the sequence of stability was RPL17>18S>EF1A>ACTB>GAPDH>TUBA>UBCE>B2M. The most stable combination of genes was RPL17 and 18S (combined stability value = 0.264). The ranking order of stability changed to B2M>TUBA>EF1A>UBCE >18S>RPL17> GAPDH>ACTB in the chemically-treated group. The most stable combination of genes were B2M and TUBA with the combined stability value = 0.039.

**Table 2:** Expression stability analysis of the 8 candidate reference genes by NormFinder.

Rank	Stages I–III	Stage I	Stage II	Stage III	Tissues	Treatment
1	UBCE	18S	GAPDH	GAPDH	RPL17	B2M
M value	0.778	0.193	0.196	0.059	0.240	0.039
2	GAPDH	GAPDH	EF1A	18S	18S	TUBA
M value	1.372	0.278	0.481	0.104	0.288	0.039
3	18S	UBCE	18S	B2M	EF1A	EF1A
M value	1.380	0.467	0.816	0.164	0.754	0.123
4	TUBA	EF1A	B2M	ACTB	ACTB	UBCE
M value	1.564	0.542	0.993	0.178	1.760	0.139
5	B2M	ACTB	UBCE	UBCE	GAPDH	18S
M value	1.827	0.712	1.008	0.203	1.973	0.22
6	EF1A	RPL17	TUBA	TUBA	TUBA	RPL17
M value	1.964	0.792	1.034	0.255	3.193	0.269
7	RPL17	B2M	RPL17	RPL17	UBCE	GAPDH
M value	2.077	0.900	1.250	0.283	3.355	1.710
8	ACTB	TUBA	ACTB	EF1A	B2M	ACTB
M value	2.376	1.123	1.958	0.765	4.331	1.823
the best	UBCE/	18S/GAPDH	GAPDH/	GAPDH/	RPL17/	B2M/
combination	GAPDH	0.154	EF1A	18S	18S	TUBA
M value	0.632		0.203	0.038	0.264	0.039

Note: The stability of the eight candidate genes, indicated by the M value were calculated by NormFinder at all developmental stages (i.e., I–III combined); as well as at developmental stage I; stage II; and stage III alone; in different tissues; and following chemical treatment.

According to the comprehensive ranking of the Ref-Finder performed, the combination of 18S and GAPDH with geomean 1.19 and 1.73 were confirmed as the most stable genes other than EF1A (2.62), RPL17 (4.76), UBCE (5.23), B2M (5.66), TUBA (5.73) and ACTB (7.74) across all developmental stages and tissue types.

### Expression Profile of LEFTY Gene of Ontogenesis and Drug Treatment

**Figure 5:**
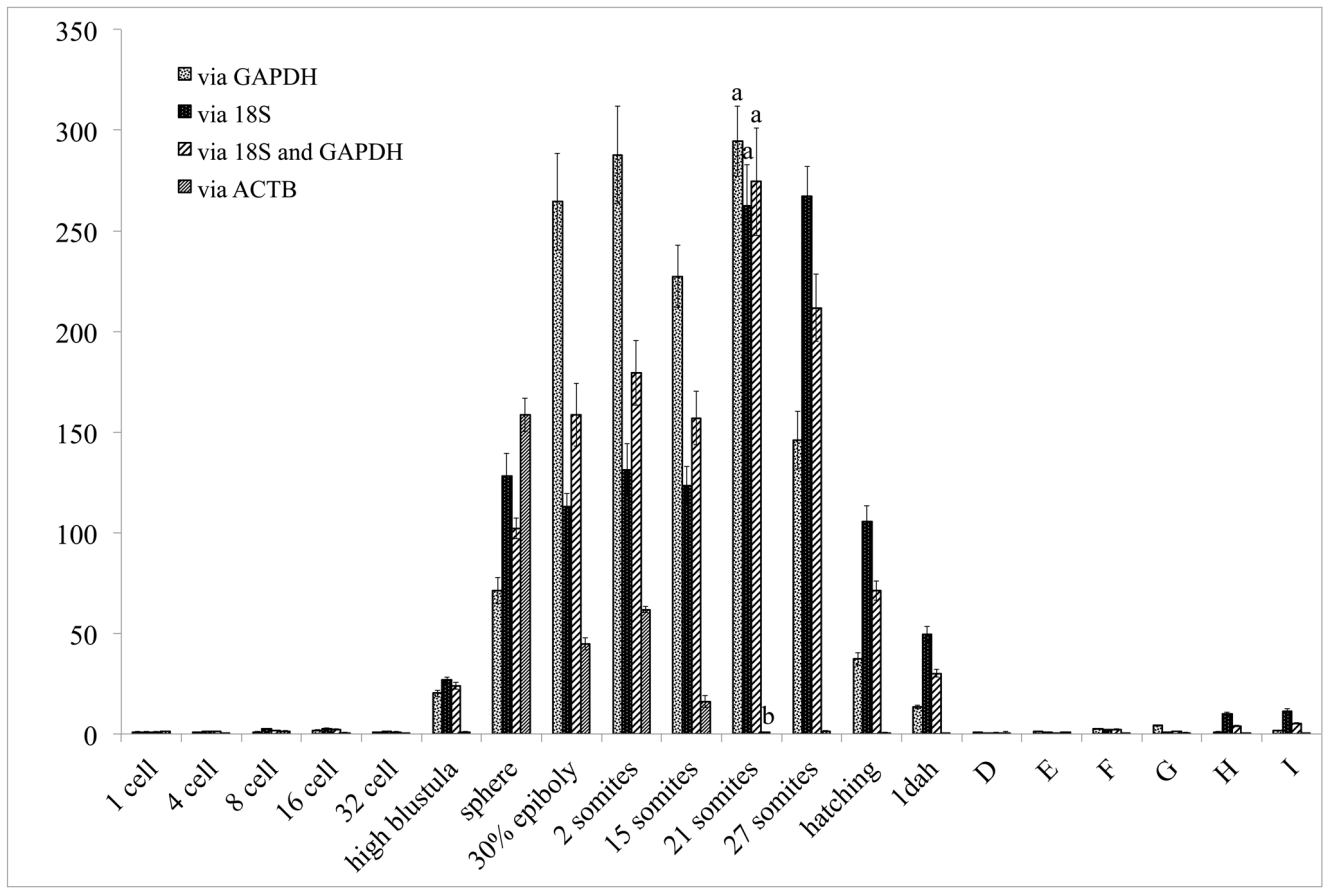
Quantitative analysis of the LEFTY gene expression in embryos. The relative expression variance is shown as the ratio between the amount of LEFTY mRNA normalized to the corresponding GAPDH values, 18S value and the geometric average of both values at a particular developmental stage and in unfertilized eggs. Different developmental stages from 1 cell stage to metamorphosis stages. Columns correspond to: 1-cell, 4-cell, 8-cell, and 32-cell stages, high blastula stage, sphere stage, 30% epiboly, as well as the 2-somite, 15-somite, 21-somite, and 27-somite stage, during hatching, in 1-day old larva, and during metamorphosis stages D∼I.

To test and verify the results of the reference gene screening, which were analyzed by geNorm, Bestkeeper and NormFinder, expression profiling of LEFTY was carried out via qRT-PCR. Four parameters, GAPDH, 18S, a normalization factor based on the geometric average of both Ct values (GAPDH and 18S) and ACTB were used to normalize the LEFTY qRT-PCR data. They showed a similar tendency during the continuous development process: a low and stable level from fertilization to the 32-cell stage, after which the level increased more or less consistently from high stage, to peak at the 21-somite stage, followed by a reduction in expression from the 27-somite stage to approximately 1 day after hatching, after which low-to-negligible levels were observed during the metamorphosis stages. However, the expression level identified by the combination of GAPDH and18S was more reliable when gene expression showed different trends between single reference gene group and the combination group. For example, LEFTY gene expression showed no significant decrease from 21 somites to 27 somites labeled by GAPDH, but it significantly dropped by the combination of GAPDH and 18S. The expression result revealed by the combination group was more consistent with the report in Japanese flounder. In addition, the expression level of LEFTY was lower compared with GAPDH/18S, and either GAPDH or 18S when ACTB was as reference gene through the entirely developmental stages. For instance, at 21 somites stage, no significant expression difference of LEFTY was shown among GAPDH, 18S and GAPDH/18S groups with high transcript abundance, but significantly lower expression was found when ACTB as reference gene (Figure 5).

## Discussion

The qRT-PCR method is widely used in gene expression studies of both freshwater [19]–[21] and marine fish [22], [23], when investigating a variety of conditions, such as infection [24], [25] and stress [20], [26]–[28], as well as in various cell lines [29]–[31]and different tissues [21]–[23], [32]. As a crucial step, the qRT-PCR data should be accurately normalized by use of the appropriate reference genes. Otherwise, the use of non-validated reference genes can lead to erroneous conclusions that are biologically meaningless [33]. However, there is no universally applicable reference gene. The same reference genes in different species and different reference genes in the same species can have dramatically different expression levels under diverse experimental conditions. For example, GAPDH has been validated as the most stable reference gene in the mandarin fish [34], but has significant expression variation in the Atlantic halibut [33] and zebrafish [35]. On the other hand, ACTB and Tubb2 showed less expression variation, but 18S rRNA and GAPDH varied significantly, even these reference genes in the same species, Atlantic halibut [36]. Thus, to improve the sensitivity and reliability of qRT-PCR in practice, the validation of reference genes should be performed for each different species, tissue type and experiment condition to be used.

The results obtained in our study were analyzed with geNorm, BestKeeper and NormFinder. The most stable reference genes generated from these three software applications showed a high level of similarity; however there were subtle deviations in the rank order. For example, throughout all developmental stages, the top 3 stable expression genes were: GAPDH, B2M and 18S for geNorm; GAPDH, 18S and B2M for Bestkeeper; and 18S, GAPDH and UBCE for NormFinder. This deviation is natural because of the different algorithms used. Both BestKeeper and geNorm use a pair-wise comparison approach, and are highly dependent on the assumption that none of the genes being analyzed are co-regulated [36]. On the other hand, in NormFinder, a separate analysis of the sample subgroups and estimation of both intra- and inter-group variation in expression levels are included into the calculation of a gene stability value [33].

In our investigation, GAPDH and B2M, were identified as the most suitable genes in samples containing all eighteen developmental stages, with an M-value of 0.57 (i.e., M <1); while18S was the next most stable gene with an M-value of 0.66 (i.e., M<1). In order to obtain a more accurate view about the stability of reference genes for the different developmental stages, we sub-divided the original eighteen developmental stages into three groups: termed I, II and III and we showed that the optimum reference genes in each group possessed M-values of less than 0.5 (Figure 3B–D). To ensure that the normalization is as accurate as possible, reference genes with the closest Ct value to that of the target genes should be selected [20]. Thus, the expression level of each reference gene was also investigated (Table S1).

According to the comprehensive ranking of the Ref-Finder performed, the combination of two reference genes, 18s and GAPDH, were shown to be the most stable genes across all developmental stages and tissue types in the half-smooth tongue sole. This is consistent with the results reported in the Atlantic salmon [37] and Japanese flounder [12]. In addition, tissue-dependent variations have been observed in the expression of most housekeeping genes in flatfish. In our study, 18S and RPL17 were shown to be the most stable reference genes. ACTB has been used previously for half-smooth tongue sole organs [38]–[40], but it was proved to be an unstable reference gene in our investigation because of significant difference between ACTB and the combination of GAPDH and 18S, even either of 18S or GAPDH. ACTB was ranked at the worst reference gene in tissues group. In contrast, ACTB/UBCE were shown to be the best reference genes in different organs of the Japanese flounder [10], and EF1A and RPSD were identified for Atlantic halibut [36] and turbot [41] tissue.

The expression stage of LEFTY was detected by *in situ* hybridization from shield-stage to the 27-somite stage in the Japanese flounder [42], We demonstrated that LEFTY appeared earlier (at high blastula, i.e., 1k-cell stage) in the half smooth tongue sole, and up to 1dpf by qRT-PCR, which is a more sensitive assay than *in situ* hybridization. In zebrafish, there are two LEFTY genes (LEFTY1 and LEFTY2). Most reports describe the expression of LEFTY1 occurring from prior to gastrulation (from sphere stage in the blastula period) [43], to the 25- or 26-somite stage in the segmentation period [44]–[49] although there is no evidence showing the expression of either LEFTY1 or LEFTY2 after this stage. However, our evidence indicates that in the half smooth tongue sole, the expression of LEFTY appears earlier and disappears later (i.e., it is expressed over a wider range of developmental stages), when compared with either the zebrafish or the Japanese flounder.

We believe that this is the first work to assess valid reference genes for qRT-PCR studies in the half-smooth tongue sole. This work is important for future developmental gene expression studies in this commercially important species as it provides valuable tools for investigating gene expression in both the embryonic and larval stages, as well as in different tissues and following chemical treatment in this flatfish species, which currently suffers from a high mortality risk during larval production.

## Supporting Information

Figure S1 **The transcriptional levels (Ct value) of 8 candidate reference genes (18S, TUBA, B2M, ACTB, EF1A, GAPDH, RPL17 and UBCE).** Presented as mean Ct value (cycle threshold value) for each candidate gene and each sample of different developmental stages, tissues and chemical treatment. For developmental stages I and II, 30 embryos were used per stage, while for developmental stage III, three larvae were used. Tissue samples were collected from three different adult fish. Experiments were performed in triplicate. (A) Embryonic developmental stages (I and II), include the 1-cell, 4-cell, 8-cell, 16-cell, 32-cell, high-, and sphere-stages, 30% epiboly, and 2-somite, 15-somite, 21-somite, and 27-somite stages; (B) Metamorphosis stages (III), includes metamorphosis stages D, E, F, G, H, I; (C) Different tissues include muscle, kidney, gill, spleen, liver, intestines, egg and sperm; (D) Embryos at the 16-cell stage were treated with SB431542 at 100 μM or 200 μM, or with DMSO or double distilled water as controls.
(TIF)

Table S1 **Groups divided according to abundance of expression of eight candidate genes.**
(DOC)

Table S2 **Descriptive statistics of 8 candidate reference genes expression based on the BestKeeper approach.**
(DOC)
