# Supplementary figures and images for: Reference Gene Selection for Quantitative Real-Time RT-PCR Normalization in the Half-Smooth Tongue Sole (Cynoglossus semilaevis) at Different Developmental Stages, in Various Tissue Types and on Exposure to Chemicals
Source: PLoS One. 2014 Mar 25;9(3):e91715. doi: 10.1371/journal.pone.0091715 (PMC3965400; doi:10.1371/journal.pone.0091715)

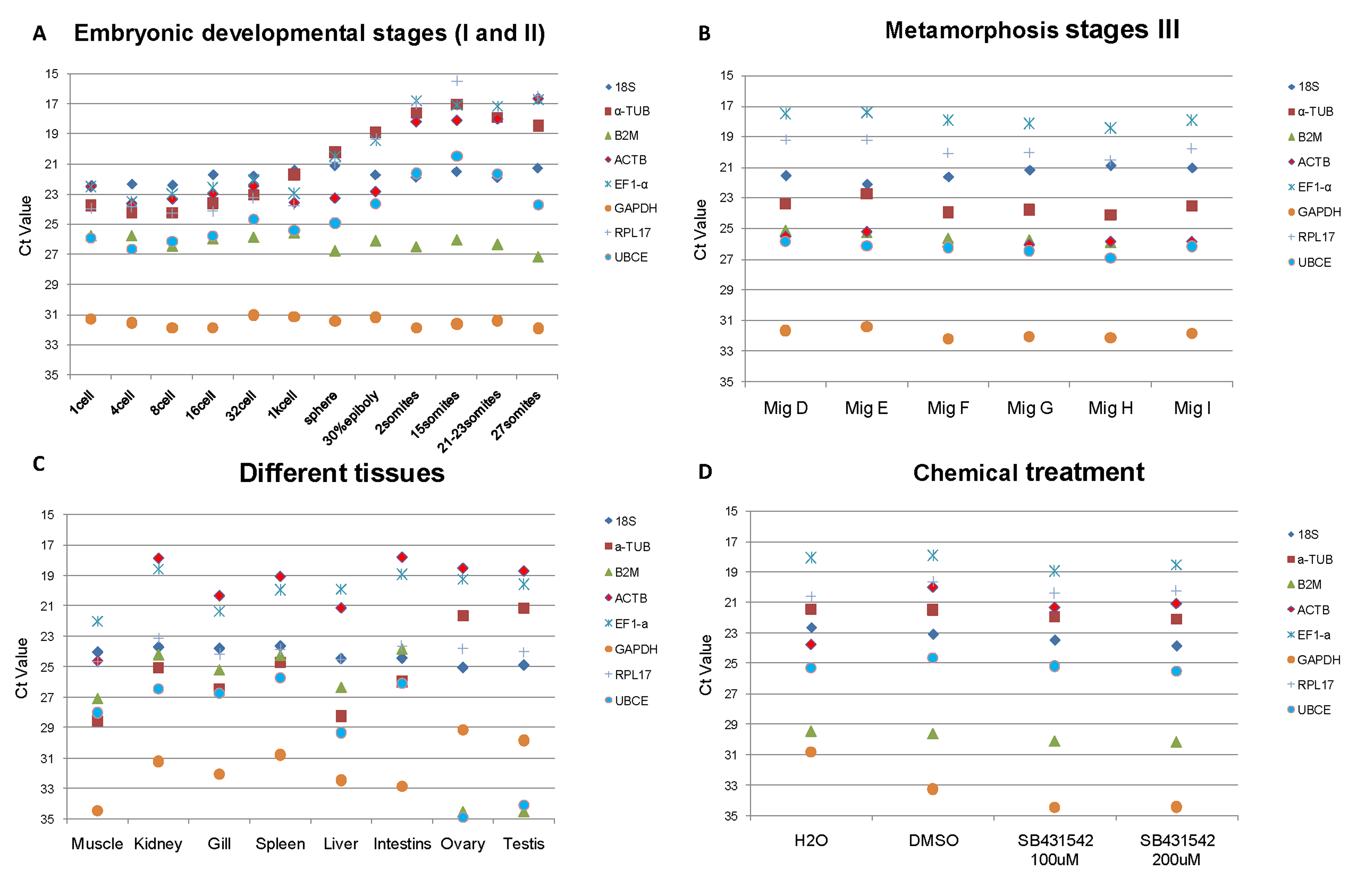

Supplement: Figure S1 — The transcriptional levels (Ct value) of 8 candidate reference genes (18S, TUBA, B2M, ACTB, EF1A, GAPDH, RPL17 and UBCE). Presented as mean Ct value (cycle threshold value) for each candidate gene and each sample of different developmental stages, tissues and chemical treatment. For developmental stages I and II, 30 embryos were used per stage, while for developmental stage III, three larvae were used. Tissue samples were collected from three different adult fish. Experiments were performed in triplicate. (A) Embryonic developmental stages (I and II), include the 1-cell, 4-cell, 8-cell, 16-cell, 32-cell, high-, and sphere-stages, 30% epiboly, and 2-somite, 15-somite, 21-somite, and 27-somite stages; (B) Metamorphosis stages (III), includes metamorphosis stages D, E, F, G, H, I; (C) Different tissues include muscle, kidney, gill, spleen, liver, intestines, egg and sperm; (D) Embryos at the 16-cell stage were treated with SB431542 at 100 μM or 200 μM, or with DMSO or double distilled water as controls. (TIF) [file pone.0091715.s001.tif]
