# Supplementary material for: Reference Gene Selection for Quantitative Real-Time RT-PCR Normalization in the Half-Smooth Tongue Sole (Cynoglossus semilaevis) at Different Developmental Stages, in Various Tissue Types and on Exposure to Chemicals
Source: PLoS One. 2014 Mar 25;9(3):e91715. doi: 10.1371/journal.pone.0091715 (PMC3965400; doi:10.1371/journal.pone.0091715)
Supplement: Table S1 — Groups divided according to abundance of expression of eight candidate genes. (DOC) [file pone.0091715.s002.doc]

Table S1 Groups divided according to abundance of expression of eight candidate genes.

|  | High transcript abundance  (average Ct value 15-20) | Median transcript abundance  (average Ct value 20-25) | Low transcript abundance  (average Ct value 30-35) |
| --- | --- | --- | --- |
| Developmental stage I | N.D* | 18S, α-TUB, ACTB, EF1-α， RPL17 | B2M, GAPDH, UBCE |
| Developmental stage II | EF1-α，α-TUB,  ACTB, RPL17 | 18S, UBCE | B2M, GAPDH |
| Metamorphosis stage III | EF1-α，RPL17 | 18S, α-TUB | B2M, GAPDH, UBCE, ACTB |
| Different tissues | EF1-α, ACTB, | 18S, RPL17 | B2M, GAPDH, UBCE, α-TUB, |
| Chemical treatment | EF1-α， | 18S, α-TUB, ACTB, RPL17 | B2M, GAPDH, UBCE |

*Note: N.D means not detected
