# Supplementary material for: Reference Gene Selection for Quantitative Real-Time RT-PCR Normalization in the Half-Smooth Tongue Sole (Cynoglossus semilaevis) at Different Developmental Stages, in Various Tissue Types and on Exposure to Chemicals
Source: PLoS One. 2014 Mar 25;9(3):e91715. doi: 10.1371/journal.pone.0091715 (PMC3965400; doi:10.1371/journal.pone.0091715)
Supplement: Table S2 — Descriptive statistics of 8 candidate reference genes expression based on the BestKeeper approach. (DOC) [file pone.0091715.s003.doc]

**Table S2 Descriptive statistics of 8** candidate reference genes expression based on the BestKeeper approach

|  |  | 18S | TUBA | B2M | ACTB | EF1A | GAPDH | RPL17 | UBCE |
| --- | --- | --- | --- | --- | --- | --- | --- | --- | --- |
| Developmental stages I-III  n=18 | geo Mean [CP] | 21.63 | 21.62 | 25.99 | 22.57 | 19.37 | 31.64 | 20.07 | 24.81 |
|  | min [CP] | 20.88 | 17.01 | 25.09 | 16.67 | 16.73 | 31.04 | 14.44 | 20.46 |
|  | max [CP] | 22.40 | 24.24 | 27.17 | 26.19 | 23.47 | 32.17 | 24.30 | 26.92 |
|  | std dev [+/- CP] | 0.37 | 2.31 | 0.38 | 2.31 | 2.25 | 0.29 | 2.47 | 1.53 |
|  | CV [% CP] | 1.71 | 10.58 | 1.47 | 10.15 | 11.52 | 0.92 | 12.18 | 6.13 |
|  | coeff. of corr. [r] | 0.807 | 0.981 | 0.733 | 0.872 | 0.905 | 0.757 | 0.945 | 0.979 |
| Developmental stages I n=7 | geo Mean [CP] | 21.84 | 22.94 | 26.03 | 23.08 | 22.39 | 31.46 | 23.42 | 25.60 |
|  | min [CP] | 21.07 | 20.22 | 25.58 | 22.45 | 20.45 | 31.04 | 20.89 | 24.63 |
|  | max [CP] | 22.40 | 24.24 | 26.77 | 23.61 | 23.47 | 31.88 | 24.30 | 26.60 |
|  | std dev [+/- CP] | 0.44 | 1.15 | 0.34 | 0.39 | 0.67 | 0.26 | 0.78 | 0.55 |
|  | CV [% CP] | 2.00 | 5.02 | 1.29 | 1.69 | 2.99 | 0.83 | 3.33 | 2.14 |
|  | coeff. of corr. [r] | 0.807 | 0.981 | 0.733 | 0.872 | 0.905 | 0.757 | 0.945 | 0.979 |
| Developmental stages II n=5 | geo Mean [CP] | 21.62 | 17.95 | 26.43 | 18.65 | 17.41 | 31.61 | 16.44 | 22.16 |
|  | min [CP] | 21.23 | 17.01 | 26.05 | 16.67 | 16.73 | 31.19 | 14.44 | 20.46 |
|  | max [CP] | 21.87 | 18.88 | 27.17 | 22.82 | 19.42 | 31.92 | 19.30 | 23.67 |
|  | std dev [+/- CP] | 0.22 | 0.56 | 0.32 | 1.62 | 0.79 | 0.24 | 1.29 | 1.15 |
|  | CV [% CP] | 1.02 | 3.10 | 1.21 | 8.66 | 4.54 | 0.76 | 7.81 | 5.20 |
|  | coeff. of corr. [r] | 0.441 | 0.179 | 0.530 | 0.779 | 0.656 | 0.256 | 0.861 | 0.530 |
| Metamorphosis stages III n=6 | geo Mean [CP] | 21.36 | 23.57 | 25.59 | 25.79 | 17.88 | 31.87 | 19.78 | 26.29 |
|  | min [CP] | 21.03 | 22.76 | 25.09 | 25.23 | 17.42 | 31.41 | 19.17 | 25.84 |
|  | max [CP] | 21.62 | 24.10 | 25.93 | 26.19 | 18.42 | 32.17 | 20.54 | 26.92 |
|  | std dev [+/- CP] | 0.19 | 0.36 | 0.28 | 0.28 | 0.29 | 0.24 | 0.42 | 0.26 |
|  | CV [% CP] | 0.88 | 1.52 | 1.08 | 1.07 | 1.61 | 0.74 | 2.10 | 1.00 |
|  | coeff. of corr. [r] | 0.822 | 0.874 | 0.644 | 0.574 | 0.806 | 0.704 | 0.900 | 0.754 |
| Different tissues n=8 | geo Mean [CP] | 24.23 | 25.09 | 27.18 | 19.67 | 19.89 | 31.57 | 24.22 | 28.72 |
|  | min [CP] | 23.63 | 21.15 | 23.82 | 17.83 | 18.59 | 29.16 | 23.13 | 25.71 |
|  | max [CP] | 25.03 | 28.53 | 34.50 | 24.64 | 21.97 | 34.46 | 25.04 | 34.88 |
|  | std dev [+/- CP] | 0.45 | 2.08 | 3.51 | 1.70 | 0.86 | 1.35 | 0.53 | 2.89 |
|  | CV [% CP] | 1.86 | 8.25 | 12.78 | 8.61 | 4.34 | 4.28 | 2.20 | 9.99 |
|  | coeff. of corr. [r] | 0.586 | 0.832 | 0.750 | 0.271 | 0.876 | 0.411 | 0.678 | 0.877 |
| Chemical treatment n=4 | geo Mean [CP] | 23.27 | 21.74 | 29.81 | 21.51 | 18.36 | 33.20 | 20.23 | 25.14 |
|  | min [CP] | 22.66 | 21.46 | 29.43 | 20.00 | 17.91 | 30.83 | 19.66 | 24.62 |
|  | max [CP] | 23.87 | 22.09 | 30.13 | 23.79 | 18.95 | 34.43 | 20.65 | 25.49 |
|  | std dev [+/- CP] | 0.40 | 0.27 | 0.30 | 1.12 | 0.37 | 1.20 | 0.29 | 0.26 |
|  | CV [% CP] | 1.71 | 1.26 | 1.00 | 5.19 | 2.04 | 3.61 | 1.44 | 1.02 |
|  | coeff. of corr. [r] | 0.461 | 0.639 | 0.586 | 0.303 | 0.753 | 0.388 | 0.651 | 0.572 |

[CP]:cycle threshold; Geo; Mean [CP]:geometric mean[CP]; Min,Max [CP]:extreme values of CP; std dev[±CP]:standard deviation[CPp]; n:number of samples; CV [%CP]:coefficient of variation (CV) based of Cp values; coeff.of corr.[r]: pairwise correlation coefficient.
